# Sick leave before and after a work-place targeted terror attack

**DOI:** 10.1007/s00420-018-1390-8

**Published:** 2018-12-06

**Authors:** Marianne Bang Hansen, Mona Berthelsen, Alexander Nissen, Trond Heir

**Affiliations:** 1 Norwegian Centre for Violence and Traumatic Stress Studies, Nydalen, Post Box 181, 0409 Oslo, Norway; 2 Institute of Clinical Medicine, Faculty of Medicine, University of Oslo, Oslo, Norway

**Keywords:** Sickness absence, Work place terror, Disaster exposure, Mental health

## Abstract

**Objectives:**

To estimate the rate of sick leave and sick leave diagnosis among employees before and after a work-place targeted terror attack, and to compare sick leave in subgroups of employees based on gender and trauma exposure.

**Methods:**

Data on sick leave and diagnosis in ministerial employees from the period 3 years before to 3 years after the 2011 bombing in the governmental district of Oslo was retrieved from the Norwegian Social Insurance Administration Registries.

**Results:**

Prior to the attack, sick leave was twice as high in women as in men. Compared to the period prior to the attack, sick leave increased the first year after the attack, for both women and men that were directly exposed to the event. Sick leave stabilized to the initial level 3 years after the incident. For indirectly exposed employees, i.e., those who were not present at the site of the attack, there was no significant increase in sick leave from before to after the attack. There were no statistical significant changes in diagnoses applied before and after the terrorist attack. However, there was a tendency towards an increase in sick leave due to psychological diagnoses among the directly exposed women.

**Conclusions:**

After a work-place terrorist attack a transient increase in sick leave may occur among employees who were present at the site of the attack. The increase may seem relatively modest and last for 1–3 years.

## Introduction

A plethora of research has established that traumatic events may provoke mental health adversities (Galea et al. 2008; McFarlane 2005; Norris et al. 2002). Systematic reviews have concluded that exposure to terrorism is associated with symptoms of anxiety and depression (Salguero et al. 2011), and posttraumatic stress symptoms (Paz García-Vera et al. 2016; Santiago et al. 2013). In many trauma populations, posttraumatic stress has been associated with comorbid mental and physical disorders (Elhai et al. 2005; Rodriguez and Kohn 2008). Post-traumatic stress is, for example, associated with hypertension and coronary heart disease (Boscarino 2008; Kibler et al. 2009), and among survivors of the 2004 Indian Ocean tsunami, musculoskeletal symptoms were reported more frequently by those severely exposed (Keskinen-Rosenqvist et al. 2011; Wahlström et al. 2013).

Sickness absence is a marker of impaired social, psychological or physical functioning for working populations (Marmot et al. 1995). In many countries long-term distress in terms of mental health problems or musculoskeletal complaints is the most common cause of sick leave (Dewa et al. 2014; Nystuen et al. 2001). The research literature on sick leave in the aftermath of disasters is rather scarce. Among Norwegian tourists who were severely exposed to the 2004 South-East Asian tsunami, 17% had long-term sick leave 6 months after the disaster, compared to 6% of those not directly exposed (Heir et al. 2010). Similarly, Swedish tourists exposed to the same event had higher sick leave due to higher levels of exposure (Wahlström et al. 2009). However, both studies refer to self-reported sick leave which may be affected by reporting bias. As far as we know, sick leave based on register data before and after a disaster has previously not been studied.

Human made disasters tend to have a longer and more severe impact on individuals than natural disasters (Norris et al. 2002). Terrorism causes extensive fear, unpredictability and loss of safety (Fullerton et al. 2003). Acts of terrorism are sometimes waged in or against a workplace or against individuals as a result of their occupation (Inness and Barling 2005). This happened on 22 July 2011 when a bomb exploded in the government district in Oslo; the ministerial employees being the target of an attack against Norwegian authorities.

The aim of this study was to estimate sick leave rates and sick leave diagnoses among employees before and after a work-place targeted terror attack. We hypothesize that women had more sickness absence compared to men, and that employees directly exposed to the bomb explosion had higher levels of sickness absence compared to those indirectly exposed. Therefore, we wanted to compare sick leave in subgroups of employees based on gender and trauma exposure.

## Methods

### Design and participants

The present study used both a prospective and retrospective cohort design to estimate sickness absence and diagnosis in ministerial employees before and after the 2011 Oslo bombing. A car bomb explosion in the executive governmental quarter shattered governmental buildings, killed eight people, and injured 209 more. This study is part of the research project “Mental health and work environment factors in the aftermath of the Oslo terrorist attack 22 July 2011” (Hansen et al. 2013).

**Fig. 1:**
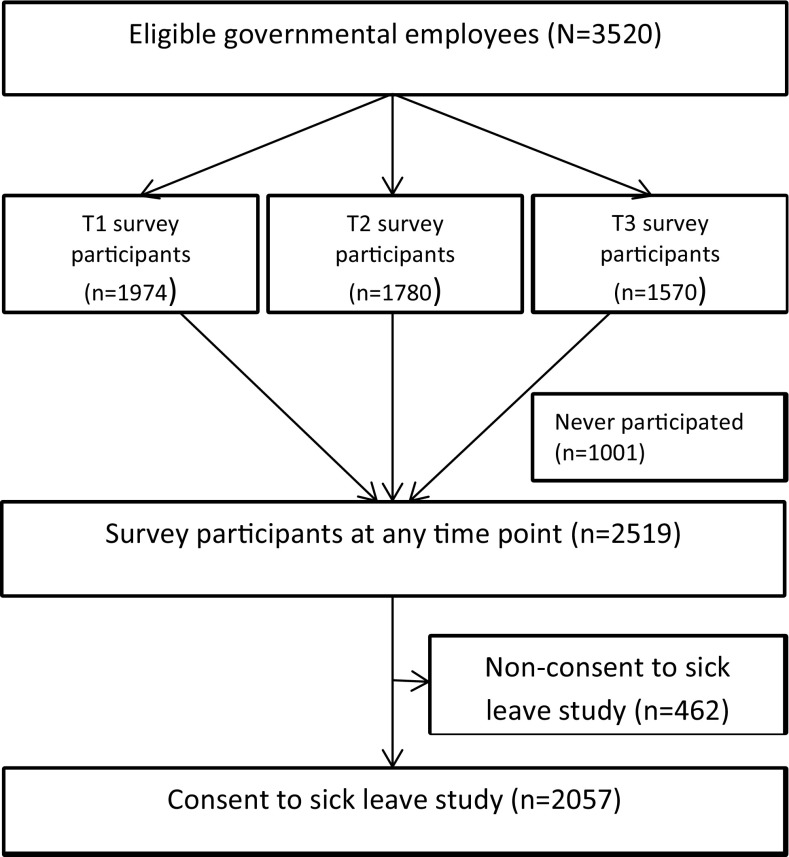
Sample overview and flow of participants

The study population comprised individuals who were employed in 14 of the 17 Norwegian ministries on the day of the terrorist attack (*n* = 3579). Of these, 59 could not be reached with information about the study. Of the remaining 3520 employees, 2519 (71.6%) participated in a Web-based survey 10, 22 or 34 months after the terrorist attack (Hansen et al. 2017). These participants were asked for permission to collect information on their sickness absence from register data, of which 2057 (82.5%) gave their consent (Fig. 1).

Prior to the Web-based surveys, a study invitation letter was sent out with information about the study, withdrawal procedures and a log-on code to access the study’s Web-based questionnaire. All employees provided written consent and strict procedures were followed to ensure confidentiality. The study was approved by the Regional committees for medical and health research ethics, REC South East, Norway.

### Data and sources of information

Data on exposure to the bomb explosion was for the participants retrieved from self-report in the Web-based survey (Hansen et al. 2017) and for the non-participants estimated from reports of total numbers given by the included ministries. Participants who were present in the governmental district when the bomb exploded were considered as directly exposed. Participants who were not present at the time of the explosion were considered as indirectly exposed. Data on education was obtained from self-report in the Web-based survey (Hansen et al. 2017). Educational level was divided into three groups: less than 13 years; 13–16 years; and more than 16 years of education.

Data on sickness absence and corresponding diagnoses was retrieved from Norwegian Social Insurance Administration Registries. Data comprised all sick leave certified by a medical doctor from January 2008 to the end of December 2014. Information included diagnosis, number of days and degree of sick leave. Data on doctor-certified sick leave in the general Norwegian working population was retrieved from the Norwegian Labour and Welfare Administration (2018).

The data on sickness absence comprised weighted sickness absence days (by percentage of full-time employment, percentage of full-time sick leave), and weighted man-days (by percentage of full-time employment, weekends, public holidays, and vacation). The number of sick days was divided by the number of man-days for each quarter, and multiplied by the possible number of man-days for the quarter. Thus, the outcome variables comprised standardized sickness absence days of full-time employment for each participant. Total sickness absence days were computed for each participant for each of the six 1-year time periods.

Each sick leave was recorded with a main diagnosis given by a physician according to the International Classification of Primary Care (ICPC) (Bentsen 1986). Variables comprising the number of days sick listed with each diagnosis were computed for all time periods. Employees with several diagnoses contributed to each diagnosis category by the number of sickness absence days due to each specific ICPC diagnosis. Diagnoses were categorized into three groups: mental disorders, diseases of the musculoskeletal system, and all other disorders.

### Statistical analyses

The number of sickness absence days represented a form of count data, characterized by substantially larger variance than the mean, a condition known as overdispersion (Cameron et al. 1999). Furthermore, the distribution of sickness absence often included more values of zeros than count values (i.e., no sickness absence). Thus, the present study employed a negative binominal hurdle model (Jackman 2010; Kleiber et al. 2008) to estimate sickness absence, which is appropriate in situations of count data with overdispersion and excess of zero-values.

The current hurdle analyses comprised (1) a binomial regression analysis which estimated the odds of having at least 1 day of medically certified sickness absence, and (2) a zero-truncated negative binomial analysis, which estimated the number of days absent among the sub-sample having at least 1 day absent. Analyses were conducted separately for the following four groups: directly and indirectly exposed females, and directly and indirectly exposed males. Analyses were conducted within four different time frames; the 1-year period before 22 July 2011 (comprising quarter three and four 2010, and quarter one and two 2011); the 1-year period after 22 July (comprising quarter three and four 2011, and quarter one and two 2012); the 1-year period 2 years after 22 July (comprising quarter three and four 2012, and quarter one and two 2013), and the 1-year period 3 years after 22 July 2011 (comprising quarter three and four 2013, and quarter one and two 2014).

The estimates from the hurdle models (four times three regressions) were then used (Zeileis et al. 2007) to compute predicted sickness absence days for each of the four groups within each of the three time periods. Adjustment was made for age (mean 45 years) and education (> 16 years). Differences in sickness absence between the four groups, and between time periods, were analyzed by computing ratios and then confidence intervals for each ratio by bootstrapping (with 10,000 bootstrap replications) using the bootstrap BC_a_ procedure. The ratios were computed within each of the three 1-year periods, for directly versus indirectly exposed females, for directly versus indirectly exposed males, for directly exposed females versus directly exposed males, and for indirectly exposed females versus indirectly exposed males. Ratios for sickness absence days were also computed for each of the four groups between time periods, i.e., sickness absence days 1, 2 and 3 years after the event versus sickness absence 1 year prior to the event.

The risk of sickness absence due to diagnosis groups was estimated separately for males and females 1 year before, 1 year after, and 3 years after the terrorist attack. The estimates from the hurdle models were then used to compute predicted sickness absence days within the diagnostic categories, for males and females, within each of the three time periods (Zeileis et al. 2007). All analyses were conducted in R, with the R packages pscl for hurdle regression and boot for bootstrap analyses (Canty and Ripley 2017; R Development Core Team 2017).

## Results

Of 2057 employees included in the study, 219 (10.7%) were directly exposed to the bomb explosion; 133 (11.6%) women and 86 (9.5%) men. Employees participating in the study had a slightly higher age and comprised relatively more women than non-participants, while their degree of exposure was quite similar (Table 1).

**Table 1:** Age, gender and proportion of ministerial employees at work (directly exposed individuals) during the 2011 Oslo bombing

	Participants (*n* = 2057)	Non-participants (*n* = 1463)
Age, mean years (SD)	45.2 (11.0)	44.0 (11.3)*
Range	20–70	19–72
Gender, *n* (%)		
Female	1149 (55.9)	744 (50.9)*
Male	908 (44.1)	719 (49.1)
Directly exposed, *n* (%)	219 (10.7)	133 (9.1)

*p < 0.01

The proportion of participants with more than 16 years of education was 68.7% (*n* = 622) for men and 61.5% (*n* = 702) for women; 22.6% (*n* = 205) men and 25.7% (*n* = 293) women had 13 to 16 years of education; while 8.7% (*n* = 79) men and 12.8% (*n* = 146) women had less than 13 years of education (*χ*^2^ = 13.54, *df* = 2, *p* = 0.001). Sickness absence among ministerial employees was lower than in the general Norwegian working population prior to, as well as after, the bomb explosion (Table 2).

**Table 2:** Doctor-certified sickness absence before and after the 2011 Oslo bombing for ministerial employees and the general Norwegian population, expressed as percentage of all working days

	25–26 mo prior	13–24 mo prior	0–12 mo prior	0–12 mo after	13–24 mo after	25–36 mo after	37–42 mo after
Ministerial employees	3.53	3.12	3.05	4.29	4.33	3.82	3.27
General Norwegian working population	6.20	6.15	5.98	5.53	5.55	5.40	5.45

*Mo* months

**Fig. 2:**
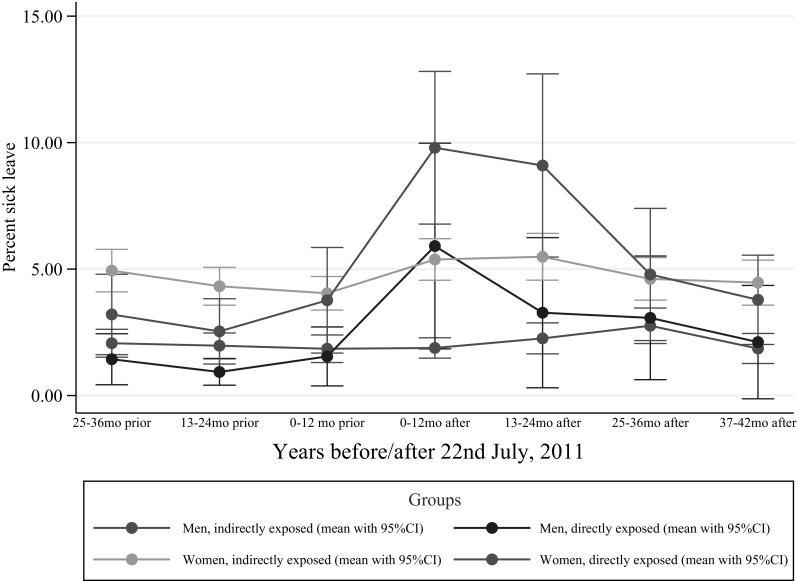
Doctor-certified sickness absence for directly and indirectly exposed female and male ministerial employees, prior to and after the 2011 Oslo bombing, expressed as percentage of all working days

Figure 2 and Table 3 show sick leave before and after the terror attack in subgroups of employees based on gender and exposure to the attack.

**Table 3:** Number of doctor-certified sickness absence days per year for directly and indirectly exposed females and males, prior to the 2011 Oslo bombing, and one, two, and three 1-year time periods after the bombing

	Directly exposed	Indirectly exposed	Exposure ratio	95% CI
0–12 months prior				
Females	7.7310	8.3419	0.9268	0.511–1.713
Males	3.2435	3.8264	0.8477	0.366–2.027
Gender ratio	2.3835	2.1801		
95% CI	0.861–6.639	**1.517–3.043**		
0–12 months after				
Females	19.5158	10.3139	1.8921	**1.318–2.650**
Males	10.8510	3.5152	3.0868	**1.430–6.530**
Gender ratio	1.7985	2.9340		
95% CI	0.801–4.072	**2.261–3.852**		
13–24 months after				
Females	18.7767	10.8915	1.7240	**1.085–2.677**
Males	6.2313	4.3568	1.4303	0.511–3.940
Gender ratio	3.0133	2.4999		
95% CI	**1.006–9.100**	**1.762–3.444**		
25–36 months after				
Females	8.6192	8.1632	1.0559	0.611–1.904
Males	5.9405	4.9729	1.1946	0.464–3.282
Gender ratio	1.4509	1.6415		
95% CI	0.481–4.717	**1.197–2.243**		

Bold values indicate a significance level of 0.05

Sickness absence ratios for gender and exposure are reported with bootstrapped confidence intervals

Women had approximately twice as high rates of sick leave compared to men in the years prior to, as well as in the years following, the attack. There was no significant difference in sick-leave rates between directly and indirectly exposed employees prior to the attack. The first year after the attack, the sick leave rates were higher in the directly exposed than in the indirectly exposed, both for men and women. In the second year after the attack, direct exposure was significantly associated with higher sick leave for women only, and in the third year there was no significant difference between those directly or indirectly exposed, neither for women nor for men.

Overall the employees had higher rates of sick-leave during the first 2 years after the terrorist attack than prior to the attack (Table 4). When split into sex and exposure, the difference was significant in directly exposed women for 2 years and in directly exposed men for 1 year, but not in women or men that were indirectly exposed (Table 5).

**Table 4:** Ratios for the entire population of ministerial employees, comparing doctor-certified sickness absence one, two, and three 1-year time periods after the terrorist attack with the last year before the attack

	Ratio	95% CI
0–12 months after/0–12 months prior	1.3124	**1.058–1.635**
13–24 months after after/0–12 months prior	1.3680	**1.082–1.749**
25–36 months after/0–12 months prior	1.1270	0.894–1.424

Bold values indicate a significance level of 0.05

**Table 5:** Ratios for directly and indirectly exposed female and male employees, comparing doctor-certified sickness absence one, two, and three 1-year time periods after the terrorist attack with the last year before the attack

	Directly exposed		Indirectly exposed	
	Ratio	95% CI	Ratio	95% CI
0–12 months after/0–12 months prior				
Females	2.5244	**1.321–4.640**	1.2364	0.967–1.582
Males	3.3454	**1.075–8.841**	0.9187	0.654–1.241
13–24 months after/0–12 months prior				
Females	2.4288	**1.154–4.542**	1.3056	0.981–1.72]
Males	1.9212	0.521–6.067	1.1386	0.782–1.695
25–36 months after/0–12 months prior				
Females	1.1149	0.503–2.326	0.9786	0.759–1.295
Males	1.8315	0.445–5.511	1.3000	0.887–1.887

Bold values indicate a significance level of 0.05

### Sick leave diagnoses

**Fig. 3:**
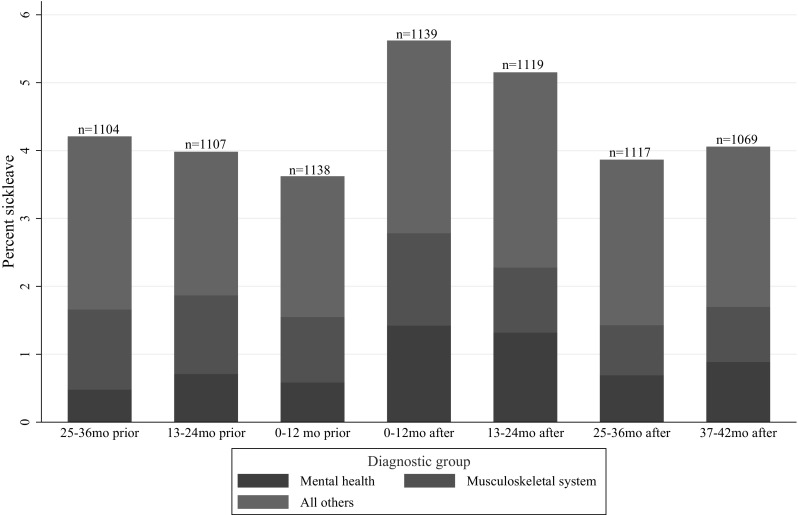
Doctor-certified sickness absence according to categories of disorders in female ministerial employees, prior to and after the 2011 Oslo bombing, expressed as percentage of all working days

**Fig. 4:**
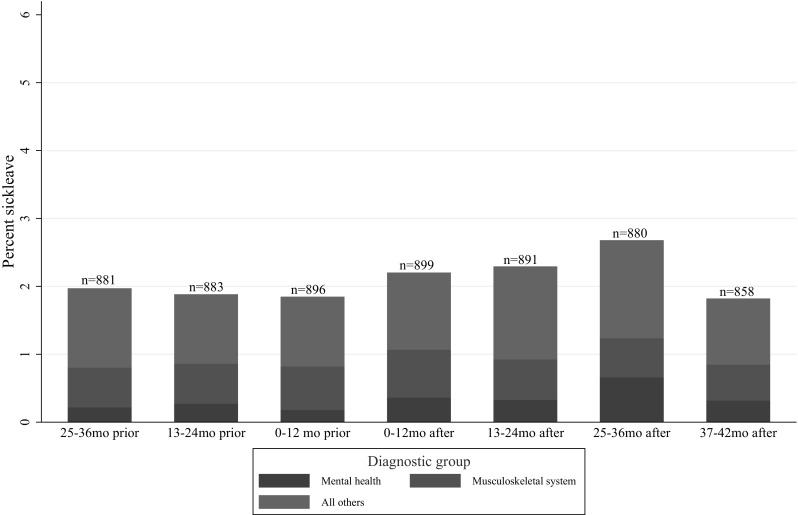
Doctor-certified sickness absence according to categories of disorders in male ministerial employees, prior to and after the 2011 Oslo bombing, expressed as percentage of all working days

Figures 3 and 4 show sick leave according to categories of physician-diagnosed disorders in women and men. There was no significant difference from before to after the disaster for any group of disorders, neither for women nor men. In women, the ratio 1-year post-attack to 1-year pre-attack was 1.71 (95% CI 0–3.54) for mental disorders and 1.63 (95% CI 0.93–3.00) for disorders in the musculoskeletal system. In men, the corresponding ratios were 1.42 (95% CI 0–4.05) for mental disorders and 1.19 (95% CI 0.54–2.48) for disorders in the musculoskeletal system. Posttraumatic stress disorder (PTSD) and depressive disorder were the most used diagnoses of mental disorders in the first 2 years after the terrorist attack both in women and men.

## Discussion

In the present study, we estimated sick leave rates and sick leave diagnoses before and after a work-place targeted terrorist attack in subgroups of ministry employees, based on gender and trauma exposure. The study revealed a transient increase in sick leave that lasted for 1–2 years in employees that were directly exposed to the terrorist attack. For those indirectly exposed, i.e., employees that were not present at the site of the attack, there was no significant increase in sick leave from before to after the attack.

An increase in sick leave was expected according to other studies of sick leave in the aftermath of traumatic events (Dirkzwager et al. 2004; Heir et al. 2010; Wahlström et al. 2009). The observed increase is most likely due to impaired health inflicted by trauma exposure. From previous research in the same study population, we have estimated that 24% of employees that were directly exposed had terror related PTSD at 10 months after the event (Hansen et al. 2013) and that the prevalence was 17% after 22 months (Hansen et al. 2017). Based on these findings, it is evident that many employees were engaged in work even though they had symptoms that were consistent with PTSD. This became even more apparent 3 years after the attack when sick leave among those directly exposed returned to levels as before the event, although the prevalence of PTSD was still 17% (Hansen et al. 2017).

Since PTSD is characterized by avoiding environments related to the traumatic event, employees who are traumatized at work often have difficulty in returning to their workplace (Stergiopoulos et al. 2011). Nevertheless, sick leave in the aftermath of this terrorist attack was far lower than, for example, among Norwegian tourists that had been exposed to the 2004 South-East Asian tsunami (Heir et al. 2010).

Some explanations should be suggested for hypothesis generation. The study population was highly educated, which is generally associated with lower rates of sick leave (Piha et al. 2010; Robroek et al. 2013). Furthermore, efforts were made to keep employees working and to help those on sick leave back to work. First, the governmental occupational health service examined the directly exposed employees for psychological symptoms, physical complaints and work ability, and offered a psychosocial follow-up program for those in need for that (Skogstad et al. 2013). Attachment to the workplace and early return to work served as key measures in the program.

Second, the occupational health service conducted a leader training program in all ministries, emphasizing the leader’s role in recognizing the severity of stress reactions and the acknowledgement of employees’ health and work effort (Weisæth and Heir 2017). A longitudinal study from before to after the terrorist attack showed that employees’ perception of leadership behavior was remarkably stable despite shortage of labor resources, implementation of relocations and rebuilding, and yet high demands on work effort (Birkeland et al. 2017).

Low statistical power due to a small number of sick leave episodes in each diagnosis group made it impossible to establish whether the increase in sick leave was associated with specific diagnoses. Apparently, there was an increase in sick leave due to mental disorders, especially among women, but also absence due to musculoskeletal complaints and other diagnoses seemed to contribute to the increase. This is consistent with research showing that posttrauma health adversities can manifest itself in a wide range of both mental and physical disorders (Elhai et al. 2005; Rodriguez and Kohn 2008).

Sick leave was roughly twice as high in women as in men prior to, as well as after, the terrorist attack. The higher sick leave rates in female employees are in line with previous research on gender and sickness absence both nationally and internationally (Bekker et al. 2009; Mastekaasa 2014). The present study adds to the field by showing that women had higher rates of sick leave than men even in the aftermath of a traumatic event that exposed women and men to the same extent. The event seemed to have greater consequence for the work ability in women, which is in line with the fact that women suffered from twice as much event-related PTSD as men (Hansen et al. 2013). The gender differences in sick leave as well as PTSD were independent of education level.

## Methodological considerations

Some limitation of the study should be noted. First, the highly educated study population may not be representative for the general working population. Second, the generalization to other countries may be questioned, as Norwegian workers are entitled to sick leave without consequences for their career or finances. Third, information about sick leave was limited to doctor-certified sick leave. Participants may have had short-term absences that were not required to be verified by a doctor, which in turn represents sick-leave not available for official calculation (Bergsvik et al. 2010). Finally, the main sick leave diagnosis selected by the physician may not reflect the diversity of the medical conditions, since several diagnoses usually contribute to each sick leave (Knudsen 2013).

Notable strengths include a large sample size, longitudinal design, and a high consent of participation. Use of registry data provided the actual number of physician-certified sick leave days, which was not subject to participants’ reporting bias.

## Implications

The present study confirms an immediate increase in sick leave in the aftermath of a work-place targeted terror attack in directly exposed employees. Rapid return to work is considered essential in posttraumatic coping (Stergiopoulos et al. 2011), and return to work and daily routines are held to be important measures to restore health, feelings of safety, trust and organizational cohesion after a work place disaster (Weisæth and Heir 2017). Organizational effort to keep employees working and to help those on sick leave back to work may have contributed to relatively low rates of long-term sick leave. The measures were directed towards the individual employee as well as the immediate leader to increase understanding and acceptance of stress responses and the need to adjust work effort. Future research should more thoroughly assess the impact of such measures. Further research should also study sick leave and possible modification effects of work environment and leadership support in the aftermath of work place disasters.
